# Practical Notes on Diagnosis and Treatment

**Published:** 1910-09-10

**Authors:** 


### Practical Notes on Diagnosis and Treatment.

### Malignant Goitre.

In malignant disease of the thyroid it is not uncommon for lymphatic enlargement to be absent, and this fact should not be allowed to weigh against the diagnosis if the other signs are suggestive. To wait for the discovery of such enlarged glands always means that the favourable time for operation has been allowed to pass.—*Mr. T. P. Legg.*

### Mitral Stenosis in Childhood.

A congenital mitral stenosis cannot be denied, but it is excessively rare. The outstanding cause is rheumatism, and that form in particular which is associated with repeated attacks of chorea. Mitral stenosis usually appears in this way: first there is a dilated heart with a mitral regurgitant bruit; then later, with repeated rheumatism, stenosis begins, and, though the lesion is a double mitral, one finds as years go by that the contraction of the orifice gains the upper hand. Mitral stenosis is rarely fatal in childhood; the lesion itself does not reach such an advanced stage before the twelfth year; there is great difference in the rate of progress in after-years.—*Dr. F. J. Poynton.*

### Aluminium Acetate.

A 1-per-cent. solution of aluminium acetate is an antiseptic of proved merit, it is entirely non-toxic, non-irritant, and markedly astringent. It is most satisfactory as a wet dressing for burns, for moist fomentations, or as the medicament for a bath in which to place an infected hand or foot for continuous irrigation, and for the washing out of suppurating cavities.—*Mr. H. F. Waterhouse.*

### Sea Bathing.

To bathe before breakfast is never free from risk, and sometimes seriously injures weakly subjects; for after the long fast of the night the circulatory and central nervous organs are more liable to depression from sudden shock or over-fatigue. On the other hand, to bathe soon after a meal arrests the process of digestion and may give rise to unpleasant gastric and cerebral symptoms. The best results are obtained from bathing two or three hours after the early morning meal, when the stomach is nearly empty, and there should be an interval of rest between the bath and the following meal.—*Dr. C. D. F. Phillips.*

